# Inhibition of PAI-1 Activity by Toddalolactone as a Mechanism for Promoting Blood Circulation and Removing Stasis by Chinese Herb *Zanthoxylum nitidum* var. *tomentosum*

**DOI:** 10.3389/fphar.2017.00489

**Published:** 2017-07-21

**Authors:** Bo Yu, Guangping Zhang, Lingling Jin, Bo Zhang, Dong Yan, Hong Yang, Zuguang Ye, Tonghui Ma

**Affiliations:** ^1^School of Life Sciences, Liaoning Provincial Key Laboratory of Biotechnology and Drug Discovery, Liaoning Normal University Dalian, China; ^2^Institute of Chinese Materia Medica, China Academy of Chinese Medical Sciences Beijing, China; ^3^College of Basic Medical Sciences, Dalian Medical University Dalian, China

**Keywords:** PAI-1, PAI-1 inhibitor, toddalolactone, fibrinolysis, pharmacology

## Abstract

Traditional Chinese medicine has been used to treat a variety of human diseases for many centuries. *Zanthoxylum nitidum* var. *tomentosum* is used as an adjuvant to promote blood circulation and remove stasis. However, the mechanisms of improving circulation and other biological activities of *Z. nitidum* var. *tomentosum* are still unclear. Plasminogen activator inhibitor-1 (PAI-1) regulates the plasminogen activation system through inhibition of tissue-type and urokinase-type plasminogen activators (tPA and uPA). PAI-1 has been linked to fibrin deposition that evolves into organ fibrosis and atherosclerosis. In the present study, we showed that ethanol extract prepared from *Z. nitidum* var. *tomentosum* exhibited PAI-1 inhibitory activity, and identified toddalolactone as the main active component that inhibited the activity of recombinant human PAI-1 with IC_50_ value of 37.31 ± 3.23 μM, as determined by chromogenic assay, and the effect was further confirmed by clot lysis assay. *In vitro* study showed that toddalolactone inhibited the binding between PAI-1 and uPA, and therefore prevented the formation of the PAI-1/uPA complex. Intraperitoneal injection of toddalolactone in mice significantly prolonged tail bleeding and reduced arterial thrombus weight in a FeCl_3_-induced thrombosis model. In addition, the hydroxyproline level in the plasma and the degree of liver fibrosis in mice were decreased after intraperitoneal injection of toddalolactone in CCl_4_-induced mouse liver fibrosis model. Taken together, PAI-1 inhibition exerted by toddalolactone may represent a novel molecular mechanism by which *Z. nitidum* var. *tomentosum* manifests its effect in the treatment of thrombosis and fibrosis.

## Introduction

Thromboembolism is a disease that can seriously affect people’s health. Especially, cardiovascular thrombosis can result in morbidity and mortality. Thrombosis is triggered by abnormal formation of blood clots in the blood stream. Fibrinolysis is a process characterized by the decomposition and liquidation of fibrin mesh formed from blood coagulation, and it involves two stages: activation of fibrinolytic enzyme and degradation of fibrin (Kivirikko et al., 1967; Morishima et al., 1997; Goto et al., 2004; Horio et al., 2007). First, prothrombin is converted to thrombin by prothrombin activator when the coagulation system is activated. Thrombin then cleaves fibrinogen and converts it into fibrin monomer, which can polymerize and form insoluble and cross-linked fibrin mesh. The anticoagulant and fibrinolytic system will also be activated along with the activation of coagulation to promote the release of plasminogen activator and fibrinolysis. The fibrinolytic system consists of plasminogen, plasminogen activators (PAs), and plasminogen activator inhibitors (PAIs). Under physiological condition, coagulation, anticoagulation, and fibrinolysis together maintain a dynamic equilibrium that ensures normal blood flow.

Plasminogen activator inhibitor-1 (PAI-1), a member of the serine protease inhibitor (serpin) gene family (Lawrence, 1997), is a key fibrinolytic inhibitor and accounts for 60% of the total PAI in the plasma. As a natural inhibitor of urokinase-type plasminogen activator (uPA) and tissue-type plasminogen activator (tPA), PAI-1 is a negative regulator of the fibrinolytic system as it suppresses the activity of PA by binding to its active site. PAI-1 is associated with a variety of thrombotic and fibrotic diseases. Overexpression of PAI-1 in transgenic mice can lead to spontaneous thrombosis, but knockout of PAI-1 in mice can protect the animals against thrombosis induced by endotoxin and chemicals (Elokdah et al., 2004; Jankun et al., 2006). As PAI-1 expression is up-regulated in thrombotic and fibrotic diseases, PAI-1 inhibition has been used in the treatment of deep vein thrombosis, coronary syndrome (Schneiderman et al., 1992), metabolic syndrome (McGill et al., 1994), cancer (Yasar Yildiz et al., 2014) and type-2 diabetes (Gorlatova et al., 2007), as well as venous thromboembolism (Crowther et al., 2001).

Identification of new small molecule PAI-1 inhibitors attracted great interest in the past decades. For example, Bryans et al. (1996) isolated two diketopiperazines, XR334 and XR330, from an unidentified *Streptomyces* species and showed that they can inhibit the interaction between PAI-1 and tPA *in vitro*, resulting in enhanced fibrinolysis and protection of the carotid artery against thrombus formation. The fibrionlytic effect of two other diketopiperazine-based low molecular weight PAI-1 inhibitors, XR1853 and XR5082, have also been evaluated in clot lysis and thrombus formation assays (Charlton et al., 1996; Crandall et al., 2004). Through these work, a novel molecule (XR5118) with improved biochemical and pharmacological profiles was obtained via chemical synthesis, and shown to inhibit thrombus accretion *in vivo* (Friederich et al., 1997). An indole oxoacetic acid derivative, tiplatinin (also named PAI-039), was subsequently prepared as a selective PAI-1 inhibitor, which can prevent carotid artery occlusion, reduce thrombus weight in vena cava while having no effect on platelet aggregation (Elokdah et al., 2004; Gorlatova et al., 2007; Hennan et al., 2008). TM5275 is another novel inhibitor of PAI-1, and its antithrombotic effect has been demonstrated in monkey model of arterial thrombosis (Izuhara et al., 2010).

In addition to these chemically defined PAI-1 inhibitors, many Chinese medicinal plants have also been used to improve blood circulation by removing blood stasis. In our previous work, we have built a library of extracts from 500 commonly used Chinese medicinal plants and screened them for PAI-1 inhibitory activity by high throughput screening. By using a chromogenic substrate-based assay, we found many fractions of these extracts with inhibitory activity against PAI-1, including one fraction from *Zanthoxylum nitidum* var. *tomentosum*, which is a variant of *Rutaceae Zanthoxylum*, mainly produced in the bushes of Pingnan area in Guangxi Province of China. The root and rhizome of *Z. nitidum* var. *tomentosum* are commonly used in folk medicine to treat traumatic injury, rheumatic, stomach pain, toothache and snakebite (Yano and Hu, 2004; Yuan et al., 2015). It is also used in conjunction with other traditional Chinese medicinal plants in the treatment of thrombosis diseases (Zhong et al., 2006). Modern pharmacological studies have shown that nitidine and ethoxychderythrine identified from *Z. nitidum* var. *tomentosum* have anti-tumor, antihypertensive, cardiotonic, antifungal and anti-inflammatory effects (Rodriguez-Guzman et al., 2011; Chen et al., 2015), but little is known about the mechanism underlying the antithrombotic and antifibrotic effects of *Z. nitidum* var. *tomentosum*. The aim of this study was to identify PAI-1 inhibitors from *Z. nitidum* var. *tomentosum* and further investigate their anti-thrombotic and anti-fibrotic effects *in vitro* and *in vivo*.

## Materials and Methods

### Animals and Chemicals

ICR mice (8–10 weeks) were fed a standard chow diet and kept under specific pathogen-free conditions at Dalian Medical University (Permit Number: SCXK liao 2008-0002).

HPLC solvents, including methanol and acetonitrile were purchased from Honeywell Burdick & Jackson corporation (United States). PAI-1 Activity Assay Kit, which includes human recombinant PAI-1, uPA and chromogenic subsrates, was purchased from Millipore Corporation (United States). Thrombin and all chemicals for polyacrylamide gel electrophoresis were obtained from Sangon Biotech (Shanghai) Co., Ltd, Toddalolactone standard was purchased from Tauto (Shanghai) Biotechnology, Co., Ltd. Rat hydroxyproline kit was bought from Nanjing Jiancheng Bioengineering Institute. The roots of *Z. nitidum* var. *tomentosum* were purchased from Beijing Tong Ren Tang, Co., Ltd. The plant sample (number A0170) was preserved in school of life sciences in Liaoning Normal University and identified by professor Yunpeng Diao (College of Pharmacy, Dalian Medical University).

### *Z. nitidum* var. *tomentosum* Extract Preparation

The roots of *Z. nitidum* var. *tomentosum* were smashed and reflux extracted with 95% ethanol for 12 h at 80°C, and the extract was then dried by rotary evaporation in vacuum to obtain a crude extract. The crude extract was dissolved in 80% methanol and separated by HPLC using a Waters autopurification system consisting a binary high pressure pump (Waters 2525), an ultraviolet visible detector (Waters 2487), an automatic collector (Waters 2767), and a preparative C18 reversed-phase column (XTerra, 19 mm × 150 mm, 5 μm particle size, United States). Methanol and water were used as the mobile phases. The separation was performed using a stepwise gradient elution as follows: 20–90% methanol in 40 min; 90–100% methanol in 10 min; and maintaining at 100% for another 5 min. The flow rate was set at 10 mL/min throughout the entire process. The eluent was monitored by absorbance at 254 nm, and fractions were collected at 6 mL per fraction. The solvent in each fraction was removed by evaporation and the residue was resuspended in DMSO to a final concentration of 5 mg/ml, followed by PAI-1 inhibitory activity assay. Peak activity fractions were then further resolved by analytical HPLC to obtain a pure compound. Analytical HPLC was carried out using an Alliance HPLC system (Waters 2695) with a photo-diode detector (Waters 2996), using analytical C18 reversed-phase column (XTerra, 2.1 mm × 150 mm, 5 μm particle size, United States).

### PAI-1 Activity Assay

PAI-1 activity assay was performed using a chromogenic assay as described previously (Loskutoff et al., 1989). Briefly, the activity of PAI-1 was determined from its inhibition of the cleavage of the colorless substrate Glu-Gly-Arg-pNA (S-2444) by uPA to generate free pNA, which generates yellow color and can be detected by absorbance at 405 nm using microplate reader (Multiskan Ascent, Thermo). One hundred microliter of reaction buffer (50 mM Tris-HCl, 150 mM NaCl, 2.5 mM CaCl_2_, 0.02% BSA, 2% DMSO, 0.1% PEG6000, pH 7.5) containing 10 nM recombinant human PAI-1 and 3 μg fraction was dispensed into a 96-well polysterene plate and incubated at room temperature for 15 min. Ten microliter of 100 nM uPA was then added to the sample followed by a further incubation for 10 min at 37°C. The proteolytic reaction was initiated by the addition of 20 μL of 2.5 g/L S-2444, and the progress of the reaction was continuously monitored at 405 nm for 30 min using Multiskan Ascent microplate reader The activity of PAI-1 was determined from the linear section or the steady state phase of the reaction, and its inhibition of uPA activity was expressed as percentage relative to control (no PAI-1).

### Clot Lysis Assay

Clot lysis assay was performed as described previously, but with slight modification (Bollen et al., 2015). Briefly, human plasma (5 μL) was mixed with 65 μL HEPES buffer (150 mM NaCl, 2 mM CaCl_2_, 20 mM HEPES, pH 7.4) in a 96-well microtiter plate, and then 10 μL human thrombin (30 NIH IU/μL) was added and maintained at 37°C for 2 h to initiate the formation of fibrin. Meanwhile, recombinant human PAI-1 (5 μM) was incubated without or with different concentrations of toddalolactone (0, 30, 60, 125, 250, and 500 μM) in a 96-well plate for 15 min at room temperature. Five microliter of 5 μM uPA was then added to each sample and incubated for another 5 min at 37°C. Each of these samples was then added to a fibrin clot generated above and the clot lysis turbidity profile was determined by measuring the absorbance at 405 nm at 5-min intervals, and over a period of 120 min. All measurements were performed in duplicate. The effect of PAI-1 on clot dissolution was expressed as percentage of clot dissolution relative to that of the control (no PAI-1).

### PAI-1/uPA Complex Formation

The effect of toddalolactone on the formation of the PAI-1/uPA complex was also determined by SDS-PAGE electrophoresis. In a 10 μL reaction volume, 1 μM recombinant human PAI-1 was first incubated with various concentrations of toddalolactone (12.5, 25, 50, and 100 μM) for 15 min at room temperature. Then, 10 μL of uPA was added to the sample (final concentration: 0.8 μM) and incubated for 10 min at 37°C. After that, 20 μL of 2× non-reduced loading buffer was added to the sample followed by heating at 100°C for 3 min. The sample was then subjected to SDS-PAGE at 80 v for 1.5 h using 10% gel. The gel was visualized by MiniBIS Pro imaging analysis system after Coomassie staining and destaining.

### Mouse Arterial Thrombosis Model

Arterial thrombosis experiments were established based on a previously described procedures (Wang and Xu, 2005) with little modifications. Firstly, mice were intraperitoneally administered saline without or with toddalolactone at a dose of 1 mg kg^-1^ for 2 weeks. Tail bleeding was performed on mice anesthetized with chloral hydrate (50 mg kg^-1^). The distal 1-mm segment was removed and the tail was immersed in 37°C distilled water. For arterial thrombosis experiments, the abdomen of the anesthetized animal was opened by a midline incision and the inferior vena artery was surgically exposed. Thrombosis was induced by applying a piece of filter paper (1 cm in diameter) saturated with 35% of FeCl_3_ to the surface of the vessel. After 30 min, a section of the vessel (∼1 cm long) was cut and the thrombus was excised. The weight of thrombus was measured after it had been washed in saline. At the end of the experiment, the artery was fixed in 10% formaldehyde, paraffin embedded, sectioned and stained with hematoxylin-eosin.

### Mouse Liver Fibrosis Model

The effect of toddalolactone on fibrosis was evaluated using a mouse liver fibrosis model induced by CCl_4_. In this experiment, the animals received a regular diet of chow, except the drinking water it received contained 5% alcohol, They were also orally administered 150 μL of CCl_4_-olive oil mixture (40% CCl_4_/60% olive oil) daily and for 7 weeks, and then intraperitoneally injected with toddalolactone at a dose of 1 mg per kg body weight for 2 weeks. As the level of hydroxyproline is an index that reflects the degree of fibrosis, the blood was collected from the eyeball, and the content of serum hydroxyproline was determined with a hydroxyproline kit. After that, the animals were sacrificed by an overdose of anesthetic, and the livers were removed and sliced into thin pieces for microscopic observation.

### Statistical Analysis

All data were expressed as means ± standard error (SE) or shown as representative traces. IC_50_ was determined by a non-linear dose-response curve fit using GraphPad Prism. One-way or two-way ANOVA followed by Dunnett’s multiple comparison tests was used to compare the test groups with the control group. Statistical significance was considered at either *P* < 0.01 or *P* < 0.05 level.

### Ethics Statement

All animals in this study were handled in accordance to the recommendations of “Guide for the Care and Use of Laboratory Animals of the National Institutes of Health,” and experimental protocol was approved by the Liaoning Normal University Committee on Animal Research. All surgical procedures were performed under sodium pentobarbital anesthesia to minimize suffering.

## Results

### Inhibition of PAI-1 by *Zanthoxylum nitidum* var. *tomentosum*

**FIGURE 1:**
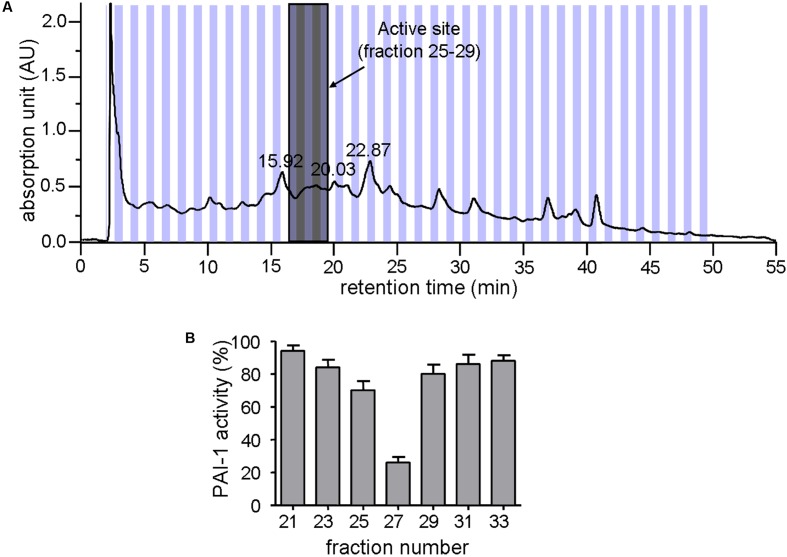
Inhibition of PAI-1 by *Zanthoxylum nitidum* var. *tomentosum* extract. **(A)** Preparative HPLC chromatogram of the extract. **(B)** Inhibition of PAI-1 activity by the HPLC-resolved fractions of the extract (*n* = 4).

*Zanthoxylum nitidum* var. *tomentosum* extract was prepared by a procedure that involved extraction with ethanol and separation by HPLC. **Figure 1A** shows the HPLC profile for the extract. Subsequent assay of the different HPLC fractions for PAI-1 inhibitory activity, which was detected by a lack of PAI-1 inhibition of urokinase activity in the presence of the extract, showed that only #25 to #29 were active, with #27 being the most active, capable of reducing the activity of PAI-1 by more than 70% (**Figure 1B**). The active fractions #25 to #29 were highlighted in gray in **Figure 1A**.

### Component Analysis and Structure Determination

**FIGURE 2:**
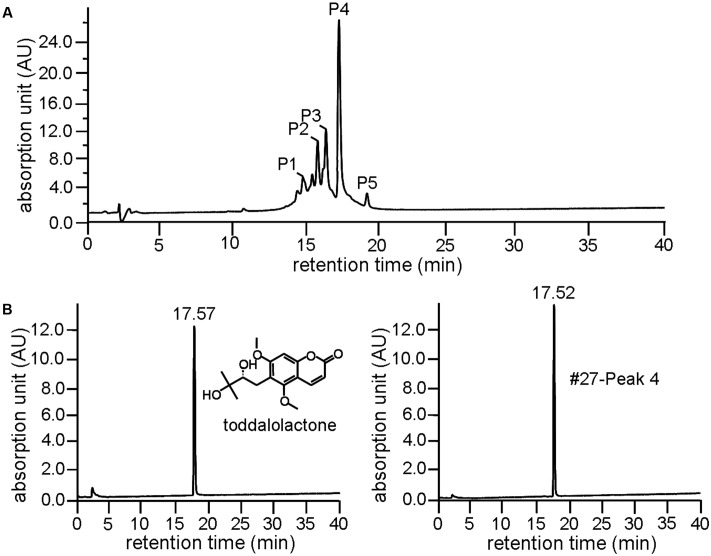
Identification of toddalolactone in the active fraction *Z. nitidum* var. *tomentosum* extract. **(A)** Analytical HPLC chromatogram of #27 with detection wavelength of 254 nm. **(B)** Chromatogram of toddalolactone standard (left) and Peak 4 of #27 of the extract (right) under the same chromatographic analysis condition. Inset: structure of toddalolactone.

Further resolution of #27 by analytical HPLC yielded several peaks with retention time between 15 and 20 min (**Figure 2A**), with Peak 4 accounting for more than 35% as determined by area normalization. Its purity was found to be 98.2%. The retention time of Peak 4 was 17.52 min, which is very close to that of toddalolactone (17.57 min, **Figure 2B**). Structural identification was further performed by comparing the ^1^H- and ^13^C-NMR spectra with those reported in the literature, and the spectral details are shown below. The compound corresponding to Peak 4 was identified as toddalolactone.

ESI-MS (m/z): 309 [M + H]^+^. ^1^H-NMR (CDCl_3_, 500 MHz) δ: 7.85 (1H, d, J = 9.65 Hz, H-4), 6.66 (1H, s, H-8), 6.25 (1H, d, J = 9.60 Hz, H-3), 3.90 (3H, s, 7-OCH_3_), 3.88 (3H, s, 5-OCH_3_), 3.60 (1H, dd, J = 10.25, 2.20 Hz, H-12), 2.93 (1H, dd, J = 13.75, 2.35 Hz, H-11a), 2.76 (1H, dd, J = 13.75, 10.30 Hz, H-11b), 2.50, 2.23 (each 1H, br s, -OH), 1.31 (3H, s, 14-CH_3_), 1.30 (3H, s, 15-CH_3_); ^13^C-NMR (CDCl_3_, 125 MHz) δ: 160.9 (C-2), 112.8 (C-3), 138.7 (C-4), 156.0 (C-5), 117.9 (C-6), 161.5 (C-7), 95.78 (C-8), 155.1 (C-9), 107.3 (C-10), 63.2 (5-OCH_3_), 56.3 (7-OCH_3_), 26.2 (C-11), 78.1 (C-12), 72.9 (C-12), 26.2 (C-14), 23.7 (C-15). Data were in agreement with the published data (Ishii et al., 1991).

### Characteristics of Toddalolactone on PAI-1 Inhibitory Activity

**FIGURE 3:**
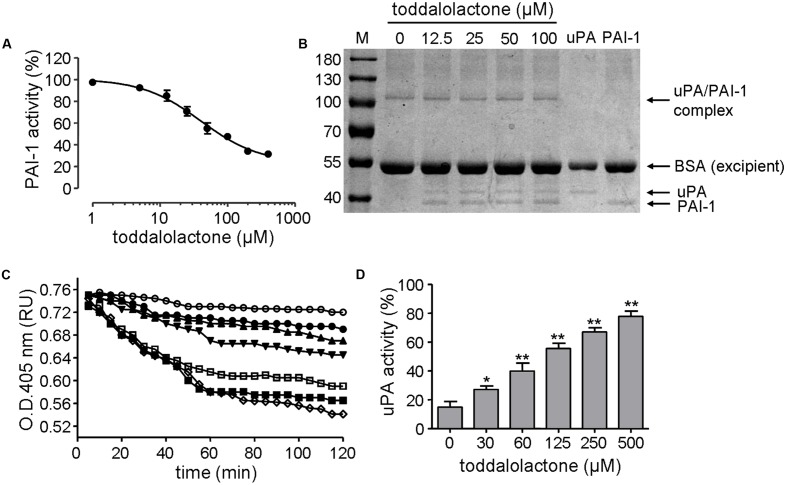
Effect of toddalolactone on PAI-1 activity. **(A)** Inhibition of PAI-1 activity by toddalolactone as determined by chromogenic assay. Data are the means ± SEs from four independent experiments. **(B)** Inhibition of PAI-1/uPA complex formation by toddalolactone. PAI-1 and uPA were incubated without or with the indicated concentrations of toddalolactone and then subjected to SDS-PAGE analysis. **(C)** Effect of toddalolactone on uPA-mediated lysis of fibrin clots. The fibrin clot was treated with uPA+PAI-1 only (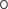) or with 30 μM (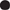), 60 μM (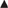), 125 μM (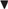), 250 μM (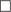) and 500 μM (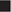) of toddalolactone, or by uPA only (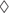). **(D)** Effect of toddalolactone on uPA activity in the presence of PAI-1. Data are the means ± SEs from five experiments, each carried out in duplicate. “^∗^” and “^∗∗^” indicate significantly different from the control (no toddalolactone) at the *P* < 0.05 and *P* < 0.01 level, respectively.

The inhibitory effect of toddalolactone on PAI-1 activity was initially evaluated by chromogenic assay. Toddalolactone inhibited the activity of recombinant human PAI-1 in a dose-dependent manner, yielding an IC_50_ value of 37.31 ± 3.23 μM (**Figure 3A**).

The mechanism of this inhibition was subsequently analyzed by SDS-PAGE. In sample containing only uPA, only one band was detected, and it had a molecular mass of about 44-kD, which is about the size of uPA. In sample containing only PAI-1, only one band with a molecular mass of about 39-kD was detected, corresponding to the size of PAI-1. However, when both uPA and PAI-1 were present in the sample, an additional band of about 105-kD appeared, which may represent the PAI-1/uPA complex (**Figure 3B**). However, the intensity of the 105-kD band appeared to decrease at higher concentration of toddalolactone, especially at 100 μM. Relatively, contents of uPA was increased slightly. The result showed that toddalolactone inhibited the formation of uPA/PAI-1 complex by preventing PAI-1 binding to uPA.

The inhibition of PAI-1 activity by toddalolactone was further demonstrated by its effect on fibrin dissolution catalyzed by uPA, in which the suppression of uPA activity by PAI-1 was relieved in the presence of toddalolactone, leading to progressive increases in fibrin dissolution (reduced OD_405_ for fibrin polymer) as the concentration of toddalolactone was increased (**Figure 3C**). This was consistent with the suppression of PAI-1 activity by toddalolactone, which enabled the activity of uPA to increase (**Figure 3D**).

### Effect of Toddalolactone on Arterial Thrombosis Induced by FeCl_3_

**FIGURE 4:**
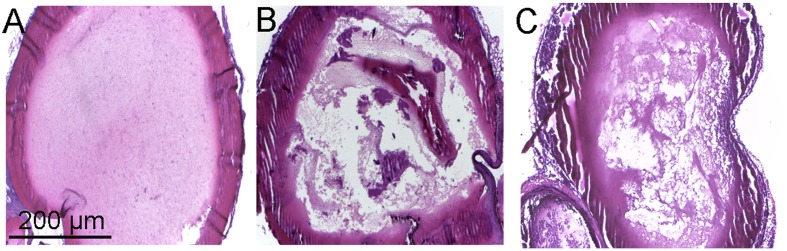
Effect of toddalolactone on arterial thrombosis induced by FeCl_3_. **(A)** Sham group; **(B)** FeCl_3_-induced mice not given toddalolactone; **(C)** toddaloctone-treated mice.

The *in vivo* antithrombotic activity of toddalolactone was evaluated by employing FeCl_3_-induced thrombotic mouse model. Compared to physiological saline (sham group), tail bleeding time in thrombotic mice given toddalolactone was increased by 54.5%. Thrombus weight was reduced by 1.6 mg after toddalolactone treatment (**Table 1**). In addition, toddalolactone also reduced the cross-sectional area of the intravascular thrombosis. In the sham group, the blood distribution and the thickness of blood vessels wall were uniform, and the structure of the vascular intima remained intact and continuous (**Figure 4A**). On the other hand, the transverse section of FeCl_3_-treated artery exhibited uneven intimal lesion, with obvious damage in all layers of the vessel wall and fibrous protein aggregation in certain particular area of the vascular lumen side, and a large area of visible thrombosis (**Figure 4B**). Administration of toddalolactone in these animals had no effect on the intimal injury, but led to a reduction of vascular wall thickness and significant reduction of thrombosis (**Figure 4C**).

**Table 1:** Effect of toddalolactone on arterial thrombosis in mice induced by FeCl_3_.

Prevention	Bleeding time (min)	Arterial thrombus wet weight (mg)
Normal saline	11 ± 3.3	8.8 ± 3.0
Toddalolactone	17 ± 2.3^∗^	7.2 ± 2.5


*Arterial occlussion evaluated by tail bleeding time and arterial thrombus wet weight. “^∗^” represents statistically significant change compared to the sham group at the ^∗^*P* < 0.05 level. Data are the means ± SEs from 6 to 8 animals.*

### Effect of Toddalolactone on Liver Fibrosis Induced by CCl_4_

**FIGURE 5:**
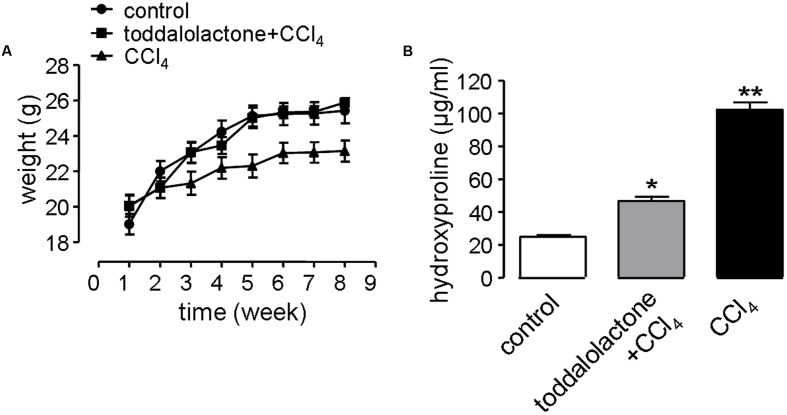
Effect of toddalolactone on liver fibrosis. **(A)** Effect of toddalolactone on weight loss in mice as a consequence of CCl_4_-induced liver fibrosis. Data are the means ± SEs from five animals. **(B)** Effect of toddalolactone on the level collagen hydroxyproline induced in mice by CCl_4_. Data are the means ± SEs from six animals. “^∗^” and “^∗∗^” indicate significantly different from the control at the *P* < 0.05 and *P* < 0.01 level, respectively.

In the liver fibrosis model, the physiological function of the nervous system and digestive system can be affected by revulsant CCl_4_. In addition, alcohol in the water can also speed up the process of liver fibrosis. Mice gavaged with CCl_4_ grew more slowly than that of control group, while after toddalolactone treatment, the body weight of the mice given CCl_4_ and alcohol was closed to normal level (**Figure 5A**).

One of the important characteristics of liver fibrosis is the large amount of collagen and extracellular matrix (ECM) secreted by hepatocytes, and hydroxyproline accounts for a high proportion of the amino acids in the collagen. Thus determining the content of hydroxyproline in the blood can reflect the level of fibrosis. The content of blood hydroxyproline in the sham group was 26.05 ± 0.7 μg/mL, however, it increased to 99.87 ± 5.1 μg/mL in the CCl_4_ model group, and the content of hydroxyproline in toddalolactone-treated group was 48.86 ± 4.3 μg/mL, which was significantly lower than that of CCl_4_-treated group (**Figure 5B**).

**FIGURE 6:**
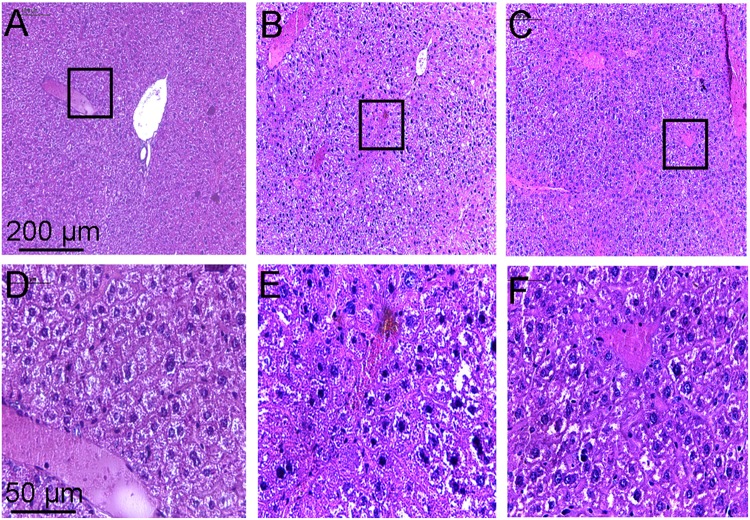
Inhibitory effect of toddalolactone on CCl_4_-induced liver fibrosis. **(A,D)** Healthy control group; **(B,F)** Model group; **(C,F)** Toddalolactone treatment group. Images in **(A–C)** are 100× magnification, whereas images in **(D–F)** are 400× magnification.

Histopathological examination of the liver showed that the hepatic lobule structure in the liver of the control mice was clear, with well-arranged hepatic cell cords, and no inflammatory cell infiltrations (**Figures 6A,D**). However, the liver of mice treated with CCl_4_ for a few weeks revealed serious disorder in the structure of lobular by paraplastic connective tissue and the hepatic cell cords, with some cellular swelling and hyperplasia of fibrous stroma being clearly visible (**Figures 6B,E**). These symptoms of the liver were ameliorated to some extent after treatment with toddalolactone (**Figures 6C,F**).

## Discussion

There are many traditional Chinese herbal plants capable of activating blood circulation and removing blood stasis, and have therapeutic effects against thrombosis and fibrosis. Examples include Ligusticum chuanxiong Hort., Salvia miltiorrhiza Bge, Gastrodia elata Bl. Whitmania pigra Whitman, etc. (Feng et al., 2012; Lu M. et al., 2015; Lu Y. et al., 2015; Liu et al., 2016). Xiao et al. (2012) identified two PAI-1 inhibitors, tanshinone IIA and cryptotanshinone, from *Salvia miltiorrhiza Bge*, and inhibition of PAI-1 was considered to be one of the mechanism by which *Salvia miltiorrhiza Bge* exerts its anticoagulant effect. Inspired by this, we speculated that PAI-1-inhibiting compounds may be an important pharmacological basis on which traditional Chinese medicines improve blood circulation. As expected, fractions isolated from the ethanol extract of *Z. nitidum* var. *tomentosum* were able to inhibit the activity of PAI-1, and a single compound was subsequently purified and identified as toddalolactone (**Figure 2B**). In the FeCl_3_-induced artery thrombosis mice model, toddalolactone significantly reduced thrombus weight and prolonged tail bleeding time, thereby providing protection against thromboembolism. Toddalolactone also reduced the severity of CCl_4_-induced liver fibrosis in mice. The study uncovered a novel anti-thrombosis and anti-fibrosis mechanism for *Z. nitidum* var. *tomentosum.*

PAI-1 is a unique serpin that can exist in two interconvertible forms: active and inactive forms. Active PAI-1 can be spontaneously converted to the latent form in a few minutes, and the half-life of the latent form is approximately 1 h (Binder et al., 2002). Additionally, substrate is another non-inhibitory form of PAI-1 which is easily cleaved by plasminogen activators (Urano et al., 1992). Thus PAI-1 can be inactivated by different mechanisms. For example, some specific antibodies of PAI-1 can prevent the formation of PAI-1/uPA complex (Bijnens et al., 2001) or facilitate active PAI-1 conversion to the latent form (Verhamme et al., 1999). Promoting PAI-1 cleavage to generate a substrate-like form can also impair uPA and tPA inhibition (Elokdah et al., 2004; Gorlatova et al., 2007). At present, many coumarin compounds have been found to have anticoagulation and thrombosis-inhibiting activities. Warfarin is a coumarin anticoagulant that has been widely used in the prevention and treatment of thromboembolic disease. Dabigatran, which is considered to be a new derivative of warfarin, might become an alternative drug in the treatment of venous thromboembolism (Bansal and Ren, 2016). A kind of coumarin derivative has been demonstrated to prevent collagen- and epinephrine-induced pulmonary thromboembolism and significantly reduce thrombus weight in arteriovenous shunt model (Jain et al., 2013). However, the exact molecular mechanisms of these antithrombotic drugs are unclear. In this study, toddalolactone as a natural coumarin inhibited PAI-1 activity by preventing the formation of a stable PAI-1/uPA covalent complex, suggesting that the inhibitory effect of toddalolactone may be due to the interference of close contact between plasminogen activator and the active center of PAI-1. Egelund et al. (2001) reported that a number of structurally distinct neutralizers can bind to a common hydrophobic area near the active site of PAI-1. By comparing the structures of different PAI-1 inhibitors, we found that most PAI-1 inhibitors belonging to the amphoteric compounds, structurally characterized by hydrophilic charged and hydrophobic groups, may play important roles in the binding of these small molecules with PAI-1 (**Table 2**). However, the lack of hydrophilic charged groups (e.g., carboxylic acid) and hydrophobic groups (e.g., aromatic groups) in the structure of toddalolactone may suggest the existence of a different mechanism that could prevent the formation of PAI-1/uPA complex.

**Table 2:** Structures and IC_50_ of several PAI-1 inhibitors.

Compound	Structure	IC_50_ (μM)	Reference
AR-H029953XX	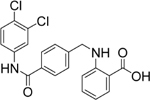	6.3	Egelund et al., 2001

XR1853	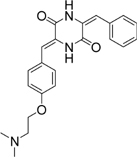	>100	Crandall et al., 2004

XR330	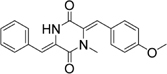	30	Bryans et al., 1996

XR334	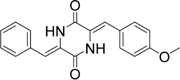	51	Bryans et al., 1996

XR5118	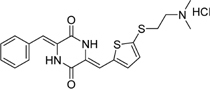	3.6	Egelund et al., 2001

TM5275	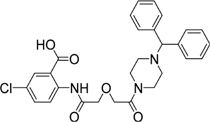	6.9	Izuhara et al., 2010

PAI-039	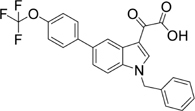	8.8	Egelund et al., 2001


The correlation between PAI-1 expression and arterial and venous thrombosis formation has been confirmed by PAI-1-overexpressing and PAI-1-deficient mouse models (Erickson et al., 1990; Carmeliet et al., 1993). In the present study, the antithrombotic effect of toddalolactone was also demonstrated in FeCl_3_-induced artery thrombosis model. After intraperitoneal administration of toddalolactone for 2 weeks, the thrombus weight and intravascular thrombosis were significantly reduced compared to the sham group, resulting in improved blood circulation and blood flow (**Table 1**). Simultaneously, the integrity of the vascular structures was protected by toddalolactone. This result was consistent with the clot lysis assay *in vitro*, which confirmed the inhibitory effect of toddalolactone against PAI-1 activity as the main mechanism of toddalolactone-mediated inhibition of thrombosis. However, this result does not rule out the possibility of inhibition of PAI-1 expression by toddalolactone.

Liver fibrosis is a common response to chronic liver injury, ultimately leading to cirrhosis and its complications. The imbalance between ECM synthesis and its degradation leads to the stimulation of ECM, which may develop into liver fibrosis, or even hepatic cirrhosis. Plasminogen activators (uPA/tPA) and plasmin play important roles in the proteolytic degradation of ECM proteins. The activities of uPA/tPA/plasmin and plasmin-dependent MMPs rely mostly on the activity of PAI-1 (Ghosh and Vaughan, 2012). Wang et al. (2007) reported that PAI-1 deficiency can increase the activity of tPA and MMP9, and reduce cholestatic liver fibrosis. In this study, the anti-fibrosis potential of toddalolactone was successfully demonstrated using a mouse model of CCl_4_-induced liver fibrosis. Long-term CCl_4_ lavage caused slow increase in the body weight of the animals. This phenomenon may be related to the activation of hepatic stellate cells and leptin secretion released in the course of liver injury, which can reduce appetite and increase energy consumption (Fried et al., 2000; Otte et al., 2004). However, the weight loss observed in the mice suffering from hepatic fibrosis was obviously reversed after toddalolactone administration. More importantly, toddalolactone could significantly reduce the collagen levels in the blood and effectively improve the leision of the hepatic tissue induced by CCl_4_.

## Conclusion

The present study identified toddalolactone as a PAI-1 inhibitor, which may contribute to the stasis-removing effect of *Z. nitidum* var. *tomentosum*.

## Author Contributions

BY designed the project, performed the experiments, analyzed the data, and wrote the manuscript. GZ, LJ, BZ, and DY performed experiments and analyzed the data. HY discussed the data. ZY and TM designed the project, analyzed, discussed the data and wrote the manuscript.

## Conflict of Interest Statement

The authors declare that the research was conducted in the absence of any commercial or financial relationships that could be construed as a potential conflict of interest.
